# Repurposing Artrestan (Sacubitril/Valsartan), unveiling its anti-inflammatory and fibrinolytic properties in ulcerative colitis in rats

**DOI:** 10.22038/ijbms.2025.87771.18964

**Published:** 2025

**Authors:** Khatereh Kharazmi, Seyedeh Elnaz Nazari, Moein Eskandari, Fereshteh Asgharzadeh, Akram Aminian, Amir Avan, Seyed Mahdi Hassanian, Majid Khazaei

**Affiliations:** 1 Department of Medical Physiology, Faculty of Medicine, Mashhad University of Medical Sciences, Mashhad, Iran; 2 Metabolic Syndrome Research Center, Mashhad University of Medical Sciences, Mashhad, Iran; 3 Department of Medical Biochemistry, Faculty of Medicine, Mashhad University of Medical Sciences, Mashhad, Iran; 4 Department of Physiology and Pharmacology, Afzalipour Faculty of Medicine, Kerman University of Medical Sciences, Kerman, Iran; 5 Medical Genetics Research Center, Mashhad University of Medical Sciences, Mashhad, Iran; 6 Molecular Medicine Group, Department of Modern Sciences and Technologies, Faculty of Medicine, Mashhad University of Medical Sciences, Mashhad, Iran

**Keywords:** Artrestan (Sacubitril/Valsartan), Fibrosis, Inflammation, Mesalazine, Ulcerative colitis

## Abstract

**Objective(s):**

Ulcerative colitis (UC) is an inflammatory disorder that is managed with various treatments, which have varying degrees of effectiveness and side effects, highlighting the need for new and more effective alternatives. In this study, we applied Artrestan (Sacubitrol/Valsartan), which has potent anti-inflammatory properties, alone or in combination with mesalazine, in the treatment of UC animal models.

**Materials and Methods:**

Thirty male rats were randomly divided into control, colitis, Artrestan (60 mg/kg/day), mesalazine (100 mg/kg/day), and Artrestan plus mesalazine groups. UC was induced by intrarectal administration of acetic acid, followed by a 5-day course of oral medication, during which the disease activity index (DAI), including diarrhea, weight loss, and rectal bleeding, was assessed daily. Macroscopic and microscopic examinations, as well as assessments of oxidant-anti-oxidant, pro-inflammatory, and pro-fibrotic factors, were performed on colonic tissue.

**Results:**

Administration of Artrestan, especially in combination with mesalazine, significantly decreased DAI and histological lesion scores in the microscopic assessment. Moreover, Artrestan modulated oxidant-anti-oxidant balance by increasing the activities of superoxide dismutase (SOD) and catalase (CAT) and reducing malondialdehyde (MDA) in colon tissue. Expression of inflammatory markers, including Interleukin 6 (IL-6) and Tumor necrosis factor-alpha (TNF-α), was decreased in the treated groups compared to the untreated group. Artrestan also attenuated fibrosis and collagen deposition in colon tissues, which was accompanied by a reduction in the expression of Transforming growth factor beta (TGF-β).

**Conclusion:**

Our findings suggest the therapeutic potential of Artrestan in combination with mesalazine for the treatment of UC, as it modulates clinical symptoms, improves the oxidant-anti-oxidant balance, and reduces pro-inflammatory and pro-fibrotic factors, supporting further investigations in the clinical phase.

## Introduction

Ulcerative colitis (UC) and Crohn’s disease (CD) are forms of Inflammatory Bowel Disease (IBD). UC is more prevalent and causes inflammation, fragility, and erosion of the colon, mainly. The main symptoms include hematochezia, diarrhea, and weight loss. UC is known for its recurring flare-ups and remissions, which can significantly impact a person’s social and professional life. One of the potential risks of UC is the susceptibility to develop toxic megacolon or colorectal cancer. Although the origin of the disease remains unknown, in affected individuals, an atypical immune response in the colon mucosa may lead to defects in the intestinal epithelial barrier, which can be attributed to the heightened production and activity of pro-inflammatory factors (1). Numerous chronic inflammatory diseases progress to tissue fibrosis, organ failure, and ultimately death (2, 3). The primary goal in managing UC is to effectively manage inflammation. Numerous studies have shown that inhibiting pro-inflammatory mediators such as IL-6 and TNF-α, and pro-fibrotic mediators like TGF-β, can also potentially alleviate symptoms of IBD (4-8). Currently, salicylates, corticosteroids, immunomodulators, and monoclonal antibodies targeting TNF-α are used to manage symptoms, while colon removal surgery, also known as colectomy, is the ultimate solution for eliminating symptoms. Unfortunately, the unpleasant side effects of these medications can discourage patients from committing to long-term treatment (1). Moreover, some medications may not provide the necessary effectiveness for successful outcomes, even when they are used properly and consistently. 

Mesalazine is the primary treatment option that plays a crucial role in assisting patients with UC to maintain remission (9). It helps reduce inflammation by inhibiting the synthesis of pro-inflammatory substances. However, the side effects of this treatment, such as headaches, nausea, and cardiomyopathy, can be concerning (10). While corticosteroids can also lead to diabetes when used long-term (11), the exorbitant expenses and limited availability of certain medications like adalimumab can also lead patients to avoid or decline treatment, worsening their condition (12). Hence, exploring alternative or complementary medicines that are more effective, simpler, and cost-efficient is essential. 

The renin-angiotensin system (RAS) helps regulate water, electrolytes, and blood pressure. Inhibiting this system reduces cytokines and factors related to inflammation and fibrosis. Angiotensin (Ang) II, an essential element of the RAS, exerts its effects on cells through AT1 and AT2 receptors. AT1 is the dominant receptor in this pathway, and increased activity in this pathway has been observed in various inflammatory conditions. Inhibition of AT1 has been found to enhance the efficiency of the AT2 receptor, which aids in reducing inflammation and fibrosis in colitis (2, 13).

Sacubitril/Valsartan or LCZ696, also known as Artrestan, is an ARNI (Angiotensin Receptor-Neprilysin Inhibitor) medication that is administered to treat heart failure. Valsartan inhibits the AT1 receptor, thereby reducing the impact of Ang II on pro-inflammatory factors and decreasing inflammation in the tissue. Furthermore, valsartan mitigates the heightened impact of angiotensin II that results from neprilysin inhibition, which in turn helps to decrease inflammation and fibrosis (14). Since neprilysin is highly expressed in the mouse intestine (14, 15), this study aimed to evaluate the therapeutic potential of Artrestan alone and in combination with Mesalazine in the treatment of UC in an animal model.

## Materials and Methods

### Animals

Thirty male Sprague-Dawley rats (230 ± 20 g) were purchased from the Pasteur Institute of Iran and placed in a well-ventilated room within the animal facility, following standard protocols (temperature: 25 ± 2 °C, humidity approximately 40%, and a 12-hour light/dark cycle). The animals had access to water and food, *ad libitum*. The standard food was obtained from the Animal Experiment Center of Mashhad University of Medical Sciences. The experiments were conducted in accordance with the university’s laboratory animal ethics protocols at its central animal house. The Experimental Animal Care Association’s Ethics Committee at Mashhad University of Medical Sciences has approved the project and assigned the code IR.MUMS.AEC.1402.074, verifying compliance with the required protocols.

### Chemicals

Mesalazine was obtained from Iran Hormone Co. (Tehran, Iran). Artrestan was purchased from Arena Life Science Co. (Tehran, Iran). Other reagents were taken from Sigma Co. (Saint Louis, MO, USA). 

### Experimental groups

**Figure 1:**
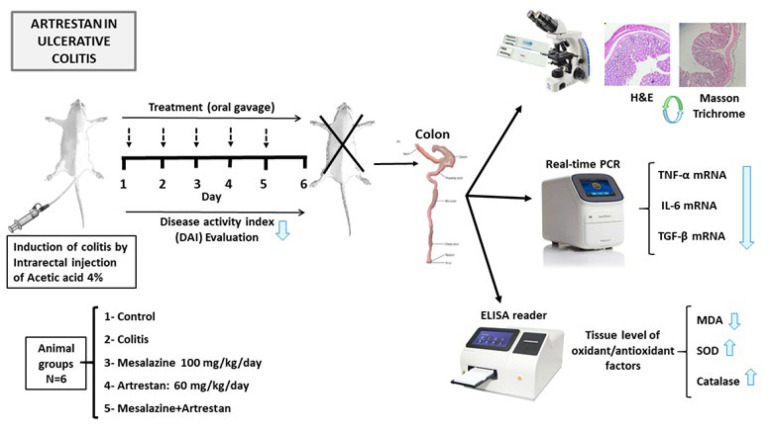
A summary of the study desgin of rat model of colitis and experimental groups

The animals were randomly divided into the following groups (n=6 each): Control: received 2 ^cc^ of N/S 0.9% by gavage and enema, Colitis with no treatment (16), Mesalazine group: received Mesalazine (100 mg/kg/day; oral gavage) (17). Artrestan group: received Artrestan (60 mg/kg/day; oral gavage) (18) and combination group: (Mesalazine+Artrestan). All treatments were initiated two hours after the induction of colitis and continued for 5 days (Figure 1). 

### Induction of experimental ulcerative colitis

For UC induction, following a 12-hour fasting and under anesthesia with ketamine and xylazine administration (40 and 5 mg/kg, respectively. IP) (19), the animals were positioned supine, subsequently, 2 ml of 4% acetic acid (v/v) was gently injected within the rectum, employing a flexible tube with a 2.7 mm diameter and 8 cm length within 30 seconds. Subsequently, the animals were immediately positioned in the supine Trendelenburg to prevent premature acid discharge. After two minutes, they were returned to the supine position to eliminate residual acid (20). Blood in rat feces or diarrhea after 24 hr suggested the development of colitis (21).

### Evaluating disease activity index (DAI)

The clinical signs of colitis were assessed using DAI, which was scored by summing the scores for weight loss, stool consistency, and rectal bleeding, as per the Cooper method. The total daily DAI score and highest DAI were reported (22, 23).

### Macroscopic evaluation of tissue damage

At the end of the study, the rats were euthanized with a high dose of ketamine and xylazine administration (300 and 30 mg/kg, respectively, IP) (24). The final 8 cm of the colon was cut, opened longitudinally, cleaned, and examined during laparotomy. Then, colon weight was measured, and tissue damage evaluation and scoring were performed using Millar’s method (25). The colon was divided into three parts for histological, biochemical, and molecular studies.

### Histopathological assessment

After fixing in 10% formalin (pH 7.4), the colon tissues were rinsed in water and then dehydrated using a series of ethanol dilutions ranging from 70% to 100%. After the steps of immersion in xylene and paraffin, the prepared microtomic tissue sections (4 µm) were deparaffinized and then rehydrated with a series of ethanol dilutions (100–70%). They were then transferred and fixed onto a slide using a special glue and subsequently stained using hematoxylin and eosin (26). Then, inflammatory changes, edema, crypt damage, and ulceration were assessed blindly by two expert specialists under a light microscope (Olympus, Japan) at ×40 and ×100 magnification. Evaluation scores ranged from 0 to 13, as detailed in Table 1 (27).

### Collagen content detection by Masson trichrome staining

The tissue sections were also stained with Masson trichrome for evaluation of fibrosis. Random images of several sites on the tissue sections were prepared from 10 high-power fields. Collagen content was estimated using ImageJ software (version 1.54, NIH) (28).

### Oxidative/anti-oxidative stress markers evaluation

The amount of oxidative and anti-oxidative stress markers, including MDA, was measured as previously described (29). In brief, tissue homogenates were mixed with thiobarbituric acid, and depending on the degree of lipid peroxidation, a pink color developed. The color intensity was then measured at 535 nm using a spectrophotometer. The activity of SOD was calculated by measuring its inhibition of the color change of pyrogallol. Then, MTT was added, and the color change was read at 570 nm (30). The activity of the CAT level in colon tissue was calculated based on the H_2_O_2_ breakdown rate, as previously reported (31). Protein concentration was determined using the bicinchoninic acid (BCA) quantification method.

### Analysis of gene expression at the molecular level (real-time PCR method)

After the extraction of total RNA, the RNA’s purity was confirmed using spectrophotometric analysis. Next, cDNA was prepared, and the amplification process was performed using PCR (RT-PCR). The sequence of forward and reverse primers is shown in Table 2. Glyceraldehyde-3-phosphate dehydrogenase (GAPDH) was considered the housekeeping gene, and all gene expression was analyzed using the 2(-Delta Delta C(T)) technique (32). R-R PCR was executed to evaluate the expression levels of three genes IL-6, TNF-α, and TGF-β, in colon tissue. 

### Statistical analysis

Data underwent analysis using SPSS statistical software (version 22). Data were analyzed using One-way ANOVA with LSD as a *post hoc* test for multiple comparisons or the Kruskal-Wallis test. Results were reported as mean ± standard error of the mean (SEM). *P*<0.05 was considered a significant level. The graphs were drawn using GraphPad Prism VIII (GraphPad Software Inc., San Diego, CA, USA).

## Results

### Effects of Artrestan administration on the disease activity index score

**Figure 2:**
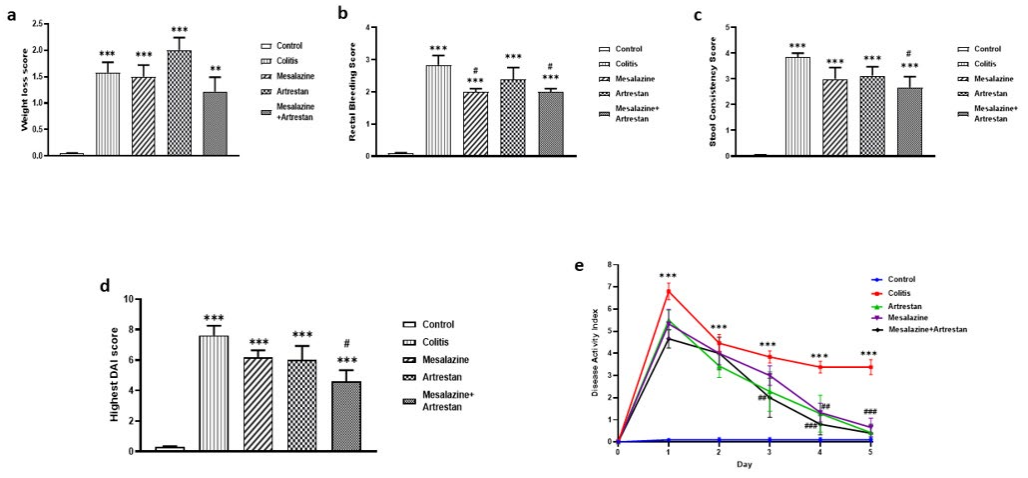
Effects of Artrestan administration on: body weight loss (a), rectal bleeding (b), stool consistency (c), DAI score at first day (d), and daily changes of DAI score period (e) in rat model of colitis

After induction of colitis and during the treatment period, DAI score, a summation of rectal bleeding and stool consistency, was measured daily. Our results showed that the colitis group experienced significantly more weight loss, rectal bleeding, and lower stool consistency scores, and had the highest DAI compared to the control group (*P*<0.001**).** Administration of Mesalazine and Artrestan improved DAI, and the combination of Mesalazine+Artrestan significantly decreased the DAI compared to the colitis group (Figure 2 a-d).

It was also found that daily changes in the DAI score were significantly higher in the colitis group compared to the control group and reduced during that time. Treatment with Artrestan and Mesalazine reduced DAI, especially in the combination group, although there were no significant differences between the treatment groups (Figure 2e).

### Colon damage and weight

**Figure 3:**
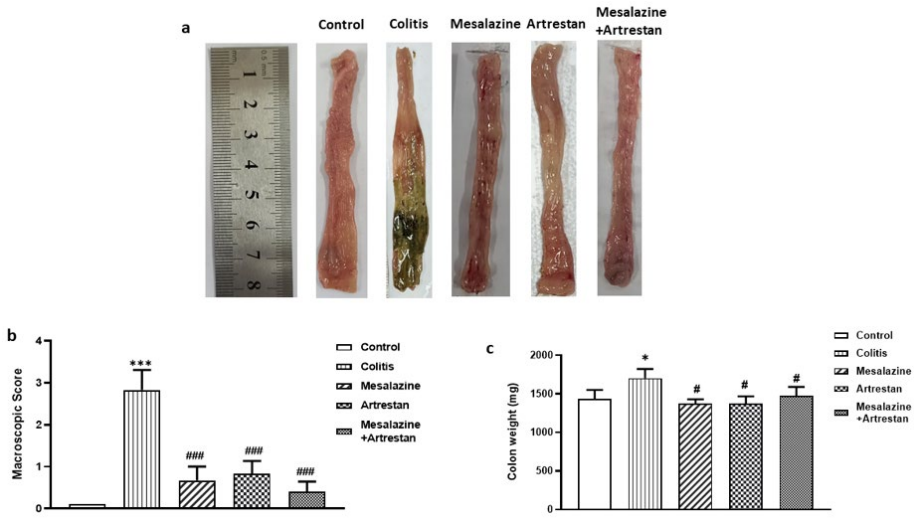
Macroscopic images of rat colon tissue in experimental groups in rat model of colitis (a), tissue damage macroscopic score (b), and colon weight (c). Data are presented as Mean ± SEM (n=6 per group)

Figure 3a illustrates the macroscopic images of the colon in experimental groups. As shown in Figure 3b, the macroscopic score of colon damage was significantly higher in the colitis group than in the control group (*P*<0.001). Treatment with Artrestan, Mesalazine, or a combination of these drugs significantly reduced this score. The colon weight was also significantly higher in the colitis group (*P*<0.0*5*). Similarly, the colon weight also decreased in the three treatment groups, with no significant changes between the treated groups.

### Histopathological findings

**Figure 4:**
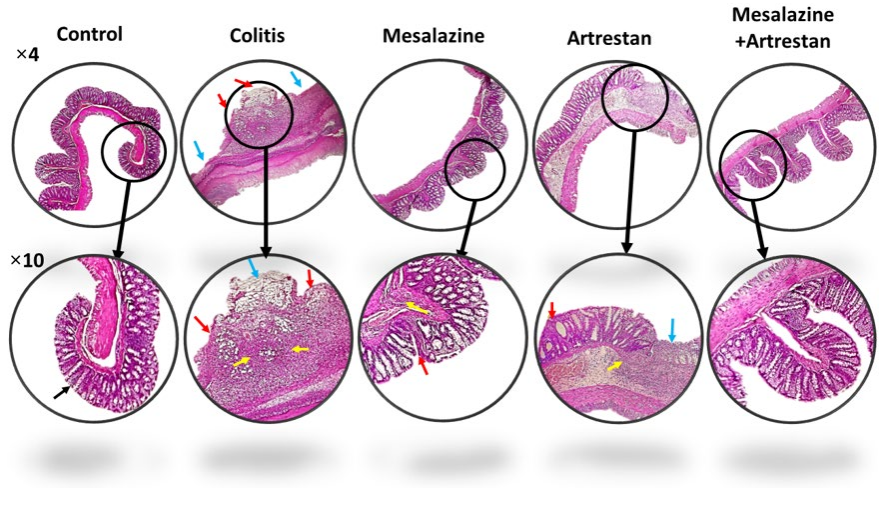
Microscopic images from the rat colons, stained by H&E method which showed more leukocyte infiltration, crypt damage and ulceration in rat model of colitis which improved by treatments especially in combination groups

**Figure 5:**
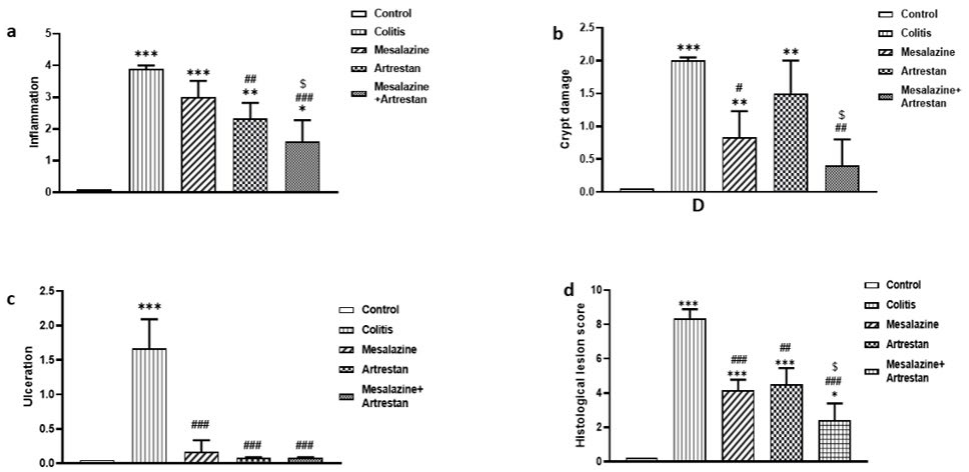
Histopathological scores from the rat colons, stained by H&E method: inflammation (a), crypt damage (b), ulceration (c), histological lesion score (d). Data are presented as Mean ± SEM (n = 6 per group)

The histological lesion score was determined by the total scores of four factors: inflammation, crypt damage, ulceration, and edema, in H&E-stained colon tissue (Figures 4 and 5, a-d). The inflammation score, assessed based on the infiltration of neutrophils in the mucosal and submucosal tissue, increased in all groups compared to the control group, with the highest level observed in the colitis group (*P*<0.001) and the lowest in the combination therapy group (*P*<0.05). The inflammation score in the Artrestan and combination therapy groups was notably lower than that in the colitis group (*P*<0.01 and *P*<0.001, respectively). However, the decrease observed in the Mesalazine group was not statistically significant.

Similarly, crypt damage scores in the colitis group were markedly higher than the control group (*P*<0.001), whereas the combination therapy group had the lowest, with no significant difference from the control group. The score was decreased in all treated groups: Mesalazine (*P*<0.05), Artrestan (*P*<0.01), and the combination therapy group, when compared to the colitis group. Additionally, the ulceration score showed a significant rise in the colitis group (*P*<0.001*) *compared to the control group. It decreased in all three treatment groups compared to the colitis group* (P*<0.001), resulting in no significant differences between any of the treatment groups and the control group. Finally, the histological lesion score in the colitis group was significantly higher (*P*<0.001), but it was reduced in all treatment groups, particularly in the combined group *(P*<0.001).

**Figure 6:**
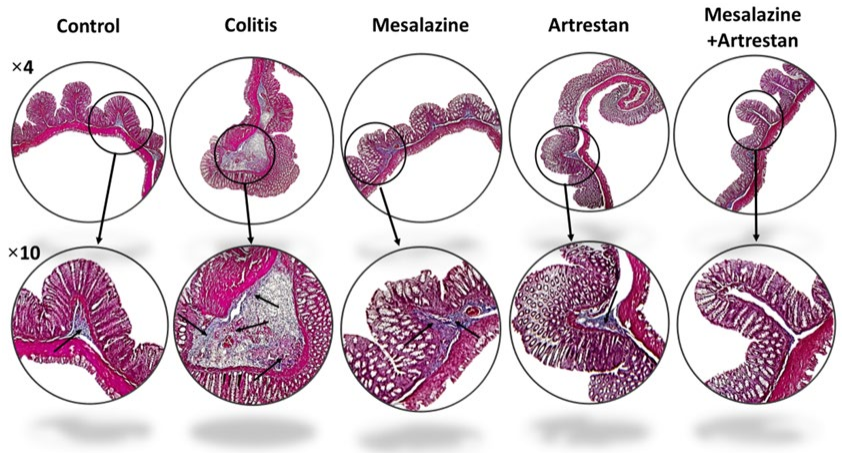
Microscopic images from the rat colons, stained by Masson’s trichrome method

**Figure 7:**
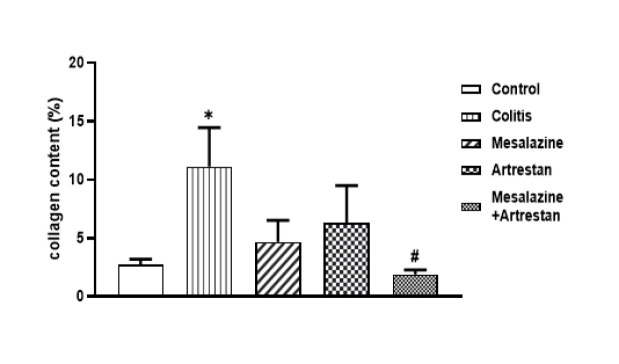
Collagen content in the rat colon, as estimated by Image J software using the microscopic images

Evaluation of Masson Trichrome-stained sections and the collagen content in the colon tissue indicated higher fibrotic tissue and collagen content in the colitis than in the control group, which significantly decreased in all treatment groups compared to the colitis group. This reduction in fibrotic tissue was more notable in the combined group, which was significantly lower than in the colitis group (Figures 6 and 7).

### Levels of oxidative and anti-oxidative stress markers

**Figure 8:**
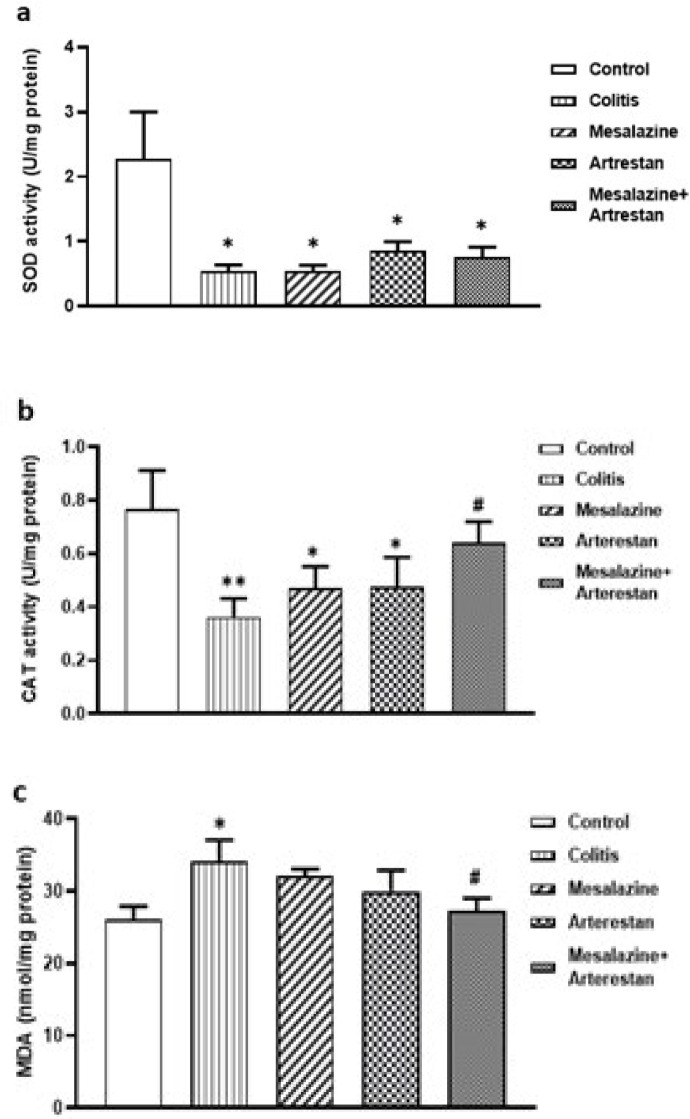
The oxidant-antioxidant factors in the rat colon, as detected by colorimetric methods: SOD (a), Catalase (b), and MDA (c)

In the assessment of oxidant-anti-oxidant factors, the colitis group exhibited a significant decrease in SOD (*P*<0.05) and CAT activity (*P*<0.01) and an elevated MDA concentration (*P*<0.05) in colon tissue compared to the control group. Treatment with Artrestan and Mesalazine resulted in higher tissue levels of CAT and a reduction in tissue MDA levels in the combination group compared to the colitis group (*P*<0.05) (Figure 8a-c).

### IL-6, TNF-α, and TGF-β expression in the colon tissue using Real-time PCR

**Figure 9:**
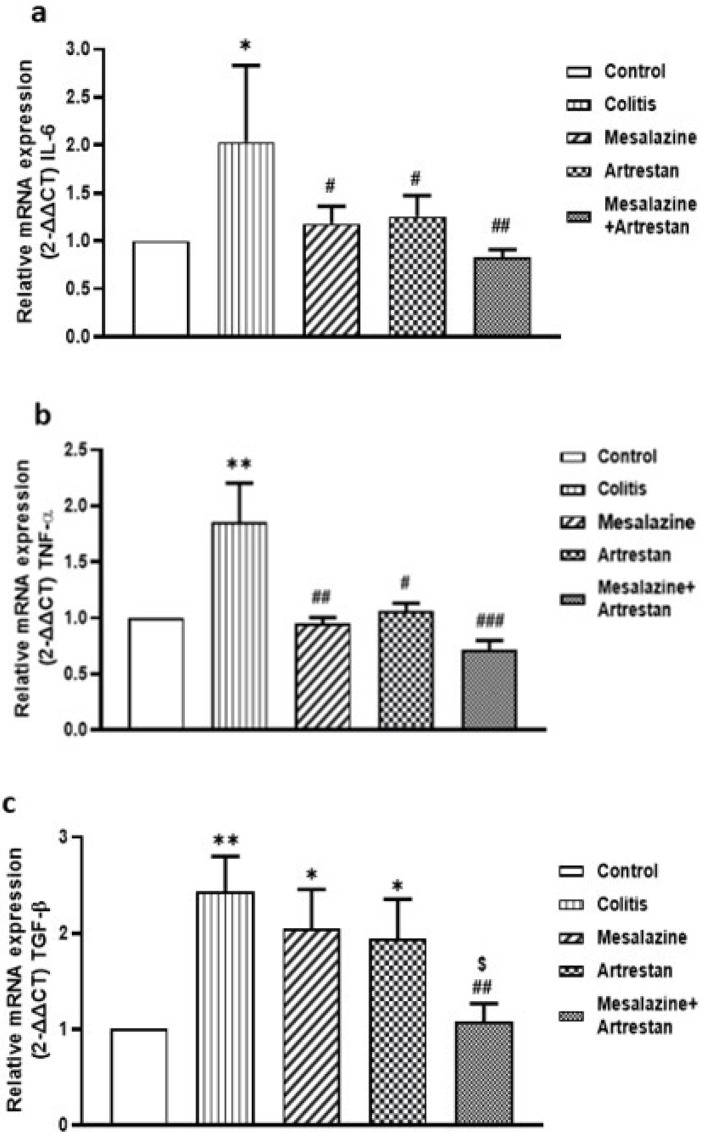
Rat colon tissue levels of mRNA expression as detected by RT-PCR method for pro-inflammatory factors: IL-6 (a) and TNF-α (b), and for pro-fibrotic factor: TGF-β (c)

RT-PCR analysis revealed that the expression levels of three genes, including IL-6, TNF-α, and TGF-β, in the colon tissue of the colitis group were higher than those of the control group. The expression of IL-6 and TNF-α genes was significantly decreased in the treatment groups, particularly in the combination group (*P*<0.01 and *P*<0.001, respectively) (Figures 9a and b). In addition, the expression of the TGF-β gene was also decreased in the treatment groups compared to the colitis group, with a significant difference in the combination group (*P*<0.01) (Figure 9c).

## Discussion

This study aimed to evaluate the therapeutic potential of Artrestan (Sacubitrol/Valsartan) in an animal model of colitis. We found that the disease activity index, including diarrhea and rectal bleeding, macroscopic score of colon damage, histopathological changes, and inflammatory and oxidative stress markers, were elevated in the colitis group, and diminished in the groups treated with Artrestan and the combination therapy of Artrestan with Mesalazine. 

The presence of diarrhea, rectal bleeding, and weight loss has been documented in numerous studies examining colitis after the colon was exposed to acetic acid in rats, and this approach also effectively replicates human-like pathological features, facilitating a more thorough investigation of disease mechanisms (33). Thus, in this study, we expected that animals exposed to acetic acid would exhibit clinical manifestations of colitis disease, such as rectal bleeding, especially in the first few days. These symptoms were confirmed by our histopathological findings, which showed increased leukocyte infiltration, crypt damage, and gross pathological changes in the colon tissue of the colitis group. Administration of mesalazine, a standard drug, improved these symptoms, which may have contributed to reducing colonic inflammation by decreasing the absorption of arachidonic acid, a precursor to prostaglandins that act as inflammatory mediators in the gastrointestinal system (34). Interestingly, we found that treatment with Artrestan, and especially the combination of Artrestan with Mesalazine, was more effective in reducing macroscopic damage to colon tissue and symptoms of colitis. A previous study indicated that the use of sacubitril/valsartan effectively promotes cell proliferation and the formation of new blood vessels, helping to reduce ischemia and increase tissue blood supply through the AT2R, which improves colon damage in these groups (18). On the other hand, inhibiting AT1R enhances the effectiveness of AT2R, which may also help reduce inflammation in this manner (35). These changes were supported by our histopathological findings, which showed reduced inflammation, crypt loss, and a lower histological score in the treated groups, with the effect being more significant in the combination group. 

We also found a notable rise in the expression of pro-inflammatory cytokines in the colons of animals with colitis. Conversely, administering Artrestan, and especially a combination of Mesalazine and Artrestan, resulted in a significant decrease in these factors. Similar to our results, studies have indicated that acetic acid-induced colitis leads to increased inflammatory cytokines, such as TNF-α and IL-6, along with other inflammatory markers, including NF-κB (36-38). On the other hand, studies have shown that sacubitril/valsartan suppresses inflammation in tissues by inhibiting TNF-α, IL-6, and NF-κB production (4-6) and reported that sacubitril/valsartan is more effective in reducing inflammatory biomarkers compared to valsartan alone. 

In the present study, the evaluation of oxidative and anti-oxidative stress markers indicated a markedly increased level of MDA and a reduced level of CAT and SOD in the colitis group, while treatment administration led to the restoration of tissue oxidative balance. Numerous studies have already demonstrated the existence of an oxidative-anti-oxidative imbalance in colitis (38, 39). In our study, treatment with Artrestane alone and in combination with Mesalazine improved the oxidative/antioxidative balance in colon tissue, offering protective benefits to tissues in the face of oxidative stress and inflammation. These results are consistent with those of a previous study, which demonstrated that oral administration of sacubitril/valsartan resulted in a reduction of oxidative factors and the restoration of tissue oxidative balance (6, 40). During the acute phase of colitis, similar to the relapse phase described in human colitis, the heightened activity of macrophages and neutrophils likely resulted in an elevated production of oxygen and nitrogen free radicals and consequently, the disruption of enzymatic functions and cellular compartments ultimately have led to damage to intestinal epithelial cells (41). All of these evidences suggest that Artrestan may have therapeutic benefits in alleviating colon inflammation. 

Finally, our results showed that, unlike the colitis group, the treatment groups, especially the combination of Artrestan and Mesalazine, exhibited a decrease in fibrotic tissue and collagen content in colon tissue. Previous studies have also revealed that colitis induced by acetic acid leads to an increase in collagen levels in the colon (38, 42), and the use of sacubitril/valsartan has resulted in decreased collagen buildup in various tissues (3, 43). Furthermore, we assessed the colonic expression of TGF-β, a well-known pro-fibrotic factor, and observed a significant decrease in the expression level of TGF-β, particularly in the combination group. Elevation of pro-fibrotic markers, including TGF-β, in colon tissue has been observed in various colitis models (7, 8, 39). Our results are consistent with several studies reporting the inhibitory effect of Artrestan on TGF-β expression and production in various tissues (44, 45).

**Table 1:** Histopathological lesion score system for colitis in rat (27)

Inflammatory cell infiltration	
0. None	
Occasional, limited to the submucosa Significant focal areas in the submucosa Significant focal areas in the submucosa and lamina propria Diffuse large areas in the submucosa, around blood vessels, and the lamina propria Transmural infiltration from mucosa to muscularis	




Crypt damage	
0. No change	
Some crypt damage, spaces between crypts Goblet cell loss, some shortening of crypts with larger spaces between them Large areas without crypts No crypt	



Ulceration	
0. None	
Small focal ulcers Frequent small ulcers Large areas without surface epithelium	


Edema	
0. Absent	
Present	
Total lesion score	0-13

**Table 2:** Rat specific qPCR primers sequence

Gene	Source	Primer	Sequence
GAPDH	Rat	Reverse Forward	CTTCCCATTCTCGCCTTGA CAACGACCCCTTCATTGACC
IL-6	Rat	Reverse Forward	TCTGGAGCCCACCAAGAACGA TTGTCACCAGCATCAGTCCCA
TNF-α	Rat	Reverse Forward	CTCTCAATGACCCGTAGGGC AGGCTGTCGCTACATCACTG
TGF-β	Rat	Reverse Forward	GGAGAGCCCTGGATACCAAC CACCCAGGTCCTTCCTAAA

**P<0.01 and ***P<0.001 vs control group; #*P*<0.05, ##*P*<0.01, and ###*P*<0.001 vs colitis group. DAI: Disease activity index

*P<0.05 and ***P<0.001 vs control group; #*P*<0.05 and ###*P*<0.001 vs colitis group.

The yellow arrows indicate leukocyte infiltration; Black arrow indicates normal crypt in colon tissue; Red arrows indicate areas of crypt damage, and the blue arrows indicate regions of ulceration.

*P<0.05, **P<0.01, and ***P<0.001 vs Control group; #*P*<0.05, ##*P*<0.01, and ###*P*<0.001 vs Colitis group; $*P*<0.05 vs Mesalazine group.

Black arrows indicate fibrotic tissue

Data are presented as Mean ± SEM (n = 6 per group). *P<0.05 vs Control group and #*P*<0.05 vs Colitis group.

Data are presented as Mean ± SEM (n = 6 per group). *P<0.05 and **P<0.01 vs Control group, #*P*<0.05 vs Colitis group.

Data are presented as Mean ± SEM (n = 6 per group). *P<0.05 and **P<0.01 vs Control group; and #*P*<0.05 vs Colitis group.

## Conclusion

Artrestan, especially in combination with a standard drug, Mesalazine, exhibits protective effects on the intestines through its anti-oxidative, anti-inflammatory, and anti-fibrotic properties, promoting and enhancing clinical symptoms and histopathological findings in colitis, and can be considered a therapeutic option for humans who have UC.

## Data Availability

Data will be made available upon request.

## Declaration

We did not use any AI tools or technologies in preparing this manuscript.
